# Comparison of anthropometric parameters and laboratory test results before and after the COVID-19 outbreak among Chinese children aged 3–18 years

**DOI:** 10.3389/fpubh.2023.1048087

**Published:** 2023-03-14

**Authors:** Wen-Hong Dong, Tian-Miao Gu, Bing-Quan Zhu, Ying Shen, Xin-Yu He, Guan-Nan Bai, Jie Shao

**Affiliations:** Department of Child Health Care, Children's Hospital, Zhejiang University School of Medicine, National Clinical Research Center for Child Health, Hangzhou, China

**Keywords:** COVID-19 lockdown, children, obesity, lipid metabolism, vitamin D deficiency

## Abstract

**Objective:**

To compare the physiological health of Chinese children around the COVID-19 lockdown.

**Methods:**

We extracted data on children's anthropometric and laboratory parameters from May to November in both 2019 and 2020 from the Health Checkup Center, Children's Hospital, Zhejiang University School of Medicine, Hangzhou, China. Overall, 2162 children aged 3~18 years without comorbidities in 2019 and 2646 in 2020 were assessed. Mann Whitney U tests were used to compare differences between the above health indicators before and after COVID-19 outbreak. Quantile regression analyses adjusted for age, sex and body mass index (BMI) were also used in analysis. Chi-square tests and Fisher's exact tests were used for comparing differences of categorical variables.

**Results:**

Compared with children examined in 2019 before the outbreak, children in 2020 had a higher median z score of BMI for age (−0.16 vs. −0.31), total cholesterol (TC, 4.34 vs. 4.16 mmol/L), low density lipoprotein cholesterol (LDL-C, 2.48 vs. 2.15 mmol/L), high density lipoprotein cholesterol (HDL-C, 1.45 vs. 1.43 mmol/L) and serum uric acid (290 vs. 282 μmol/L), and a lower hemoglobin (Hb, 134 vs. 133 g/L), triglycerides (TG, 0.70 vs. 0.78 mmol/L) and 25(OH)D (45.8 vs. 52.2 nmol/L), all *P* < 0.05. No differences were identified for waist height ratio, blood pressure and fasting glucose (both *P* > 0.05). However, in regression models after adjusting, BMI, TC, LDL-C, blood glucose and sUA were positively correlated with year; while Hb, TG and 25(OH)D were negatively correlated with year (all *P* < 0.05). Accordingly, children in 2020 had a higher prevalence of overweight/obesity (20.6 vs. 16.7%, *P* < 0.001), hypercholesterol (16.2%vs. 10.2%, *P* < 0.001), high LDL-C (10 vs. 2.9%, *P* < 0.001), hyperuricemia (18.9 vs.15.1%, *P* = 0.002), vitamin D deficiency (22.6 vs. 8.1%, *P* < 0.001) and a lower prevalence of high TG (4.3 vs. 2.8%, *P* = 0.018) compared with children in 2019.

**Conclusion:**

In this real-world study, we found that long-term lockdown due to COVID-19 outbreak might cause adverse impact on children's metabolic health, which might increase their future risk of cardiovascular diseases. Thus, parents, health professionals, educationists, and caregivers should pay more attention to children's dietary pattern and lifestyle, especially in this new normal against COVID-19.

## Introduction

In December 2019, a global health crisis of a highly infectious respiratory disease caused by SARS coronavirus 2 (SARS-CoV-2), also known as Coronavirus Disease 2019 (COVID-19) broke out worldwide (1). According to WHO's latest situation report, more than 754 million individuals were confirmed cases of COVID-19, and over 6.8 million deaths were attributable to the disease (2). Due to the severity of the epidemic and changing variants of the virus, rigorous and intensive measures are taken globally to control the transmission and mortality of COVID-19. Though social distancing and restriction measures such as home quarantine, travel ban, and even lockdown of the whole city have effectively suppressed the spread of COVID-19 (3), the potential adverse impacts on the individual's health, especially on children's health warrants more attention.

People may change their dietary and lifestyle during the COVID-19 lockdown period (4–6). In an Italian survey (4, 5), the youths had lower adherence to the Mediterranean diet compared with people aged 18–30 years old, and 70.5% of participants reported a reduced level of physical activity. In a study performed among 3,052 adults in the United States (6), social isolation induced the largest reduction in physical activity and significant increase in sedentary and screen time among adults who were previously physically active.

Similarly, children who were asked to stay at home and study online also experienced significant changes of lifestyle (7–9). In China, all kindergartens and schools were closed from January to the end of Autumn in 2020 during the lockdown (10), which may resulting lifestyles changes in three aspects. First, nutrition-balanced food provided by schools was discontinued and diverse family-cooked food was provided to children during the lockdown. Second, regular outdoor physical activity in school days were canceled after school closure. Third, children and adolescents were asked to study online at home, more screen time and more sedentary lifestyle are inevitable (7–9). However, how would the long-term COVID-10 lockdown influence children's physiological health, especially objective indicators of health such as anthropometric parameters and laboratory biochemical results, remains unknown.

To address the afore-mentioned issue, we therefore used real-world data extracted from the electronic health records from the Health Checkup Center of Children's Hospital, Zhejiang University School of Medicine, to investigate the impact of COVID-19 lockdown on children's physiological health, including bodyweight, blood pressure, fasting blood glucose, 25(OH)D, and lipid indicators, etc.

## Materials and methods

### Study design and participants

The present study was a retrospective study. Considering that the health impacts of COVID-19 lockdown would not take effects soon after the beginning of lockdown, and would not disappear the moment school re-opened, we extracted data between May (3 months after the beginning of lockdown) and November (3 months after the re-open of schools) in 2020 from the database of the Health Checkup Center. For comparison, data from the same period in 2019 was also retrieved. In analysis, children who stayed at home constantly regardless of the pandemic (aged <3 years), or had missing data on basic physical examination records such as height or weight, or with self-reported history of chronic diseases that would prohibit them from attending schools and being active regularly such as cancer, congenital heart disease, genetic metabolic disease, or diabetes mellitus were excluded. Eventually, 2,162 children from 2019 and 2,646 from 2020 were included. The study was approved by the Medical Ethics Committee of Children's Hospital, Zhejiang University School of Medicine [review number: 2021-IRB-185]. Since data used in this study was retrieved from electronic records in the hospital, and no additional physical harm were added to the participants due to this study, informed consent was waived by the Medical Ethics Committee.

### Measurements of health indicators

Children's age, sex, anthropometry parameters and laboratory test results were extracted from the hospital information system. Anthropometric parameters including height, weight, waist circumference, and blood pressure were measured by well-trained nurses. Blood samples were sent to the Department of Clinical Laboratory within 1 h after collection and tested according to established standards. Hemoglobin (Hb) was determined by colorimetry (Mindray BC-5310, Shenzhen, China); blood glucose and triglycerides (TG) was analyzed using hexokinase method and glycerol phosphate oxidase-peroxidase method, respectively (Beckman Coulter AU5800, USA); enzymatic color test methods were used for quantification of total cholesterol (TC), low density lipoprotein-cholesterol (LDL-C), and serum uric acid (sUA) (Beckman Coulter AU5800, USA). 25(OH)D was tested manually using ELISA (ImmnoDiagnosticSystem, UK). Results of all above-mentioned laboratory results were retrieved.

Children's weight status was evaluated by the Z score of BMI for age (zBMI). For children under 5 years, zBMI ≤-2, −2 <zBMI ≤2, 2 <zBMI ≤3 and zBMI>3 were defined as underweight, normal weight, overweight (OW) and obesity (OB), respectively. For children older than five, zBMI ≤-2, −2 <zBMI ≤1, 1 <zBMI ≤2 and zBMI>2 were defined as underweight, normal weight, OW and OB, respectively (11). According to Meng's study (12), waist to height ratio (WHtR)>0.48 was referred as central obesity. Hypertension was defined if systolic and/or diastolic blood pressure was higher than 95% of Chinese children of the same sex and age (see Table 1) (13).

**Table 1:** The 95 percentiles of age- and sex-specific values of blood pressure for Chinese children.

**Age, years**	**Male**		**Female**	
	**SBP, mmHg**	**DBP, mmHg**	**SBP, mmHg**	**DBP, mmHg**
3~	105	69	104	68
4~	107	70	105	69
5~	110	72	107	71
6~	112	74	110	73
7~	115	77	112	75
8~	117	78	115	77
9~	119	79	117	78
10~	120	80	118	80
11~	122	81	121	80
12~	124	81	122	81
13~	125	82	123	81
14~	127	83	123	82
15~	129	84	123	82
16~	130	85	123	82
17~	132	85	124	82

SBP, systolic blood pressure; DBP, diastolic blood pressure.

According to the WHO criteria, anemia was defined when Hb ≤110 g/L for children under 5 years' old; for children aged 5~11 years, anemia was defined when Hb ≤115g/L; for children aged 12~14 years and girls older than 15 years, Hb ≤120g/L was anemic; and for boys older than 15 years, Hb ≤130g/L was defined as anemia (14). Vitamin D status was categorized as adequate (serum 25(OH)D >50.0 nmol/L), insufficiency (>37.5 and ≤50.0 nmol/L) and deficiency ( ≤37.5 nmol/L) (15). TG≥1.70 mmol/l, TC≥5.18 mmol/L, LDL-C≥3.37 mmol/L, and HDL-C <1.04 mmol/L would be hypertriglyceridemia (hTG), defined as hypercholesteremia (hTC), high LDL-C (hLDL-C), and low HDL-C (lHDL-C) according to the Chinese Experts' Consensus (16). Since there is a lack of uniform standards for hyperuricemia (hUA), sUA above 357 μmol/L was defined as hyperuricemia, as illustrated in an U.S. National Health and Nutrition Examination Survey finding (17).

### Statistical analysis

Continuous variables were described as Median and interquartile ranges since they were non-normally distributed; categorical variables were presented as numbers and percentages. The Mann Whitney U test was used to compare differences in health indicators between 2019 and 2020. Quantile regression models were applied to assess the independent influence of lockdown after the COVID-19 outbreak on children's health indicators by adjusting for children's age, sex and BMI when appropriate. We have also transferred continuous variables into categorical variables to understand the prevalence of OB, abdominal obesity, dyslipidemia, and vitamin D deficiency in children before and after the COVID-19 outbreak. Chi-square tests were used for detecting differences in the prevalence before and after the outbreak, and Fisher's exact tests were used when needed. All analyses were performed using SAS 9.2 and a two-sided *P* < 0.05 was considered as statistically significant.

## Results

Table 2 presents the physical measurements and laboratory test results of the study population. In total, 2,162 children (60.5% males) aged 3–18 years between May and November in 2019 and 2,646 children (60% males) from the same period in 2020 were compared and children who were examined in 2020 were slightly younger (*P* = 0.001). Compared with children examined between May and November in 2019, children examined in 2020 had a higher zBMI and HDL-C (both *P* < 0.05), especially in boys (both *P* < 0.05) than their counterparts examined in 2019. Higher TC, LDL-C, HDL-C and sUA level and lower hemoglobin, TG and 25(OH)D levels were observed in children examined 2020 (all *P* < 0.05). No significant difference of WHtR, SBP, DBP, or fasting glucose levels was observed (*P* > 0.05).

**Table 2:** Physical measurements and laboratory test results before and after COVID-19 outbreak among children aged 3~18 years' old.

		**Year**				
**Characteristics**	**2019**		**2020**		***P***
	***N*** **(**n*/%)	**M (P25–P75)**	***N*** **(**n*/%)	**M (P25–P75)**	
Total	Age, years	2,162 (0)	8.9 (6**–**12)	2,646 (0)	8.3 (5.7**–**11.8)	**0.001**
	Males, yes	1,307 (60.5)	/	1588(60)	/	0.758
	zBMI	2,612(0)	−0.31 (−1.07**–**0.59)	2,646 (0)	−0.16 (−0.9**–**0.82)	**<0.001**
	WHtR	2,121 (41)	0.43 (0.4**–**0.47)	1,784 (862)	0.43 (0.4**–**0.47)	0.129
	SBP, mmHg	2,080 (82)	101 (93**–**110)	2,579 (67)	101 (93**–**109)	0.416
	DBP, mmHg	2,080 (82)	62 (56**–**69)	2,579 (67)	62 (55**–**68)	0.136
	Hb, g/L	2,146 (16)	134 (128**–**141)	2,508 (138)	133 (127**–**139)	**<0.001**
	TG, mmol/L	1,672 (490)	0.78 (0.62**–**1.04)	2,020 (626)	0.70 (0.55**–**0.95)	**<0.001**
	TC, mmol/L	1,672 (490)	4.16 (3.73**–**4.68)	2,020 (626)	4.34 (3.89**–**4.87)	**<0.001**
	LDL-C, mmol/L	1,603 (559)	2.15 (1.85**–**2.52)	1,983 (663)	2.48 (2.12**–**2.88)	**<0.001**
	HDL-C, mmol/L	1,603 (559)	1.43 (1.26**–**1.62)	1,983 (663)	1.45 (1.28**–**1.65)	**0.007**
	25(OH)D, nmol/L	1,674 (488)	52.2 (45.2**–**60)	1,880 (766)	45.8 (38.4**–**57.1)	**<0.001**
	Fasting glucose, mmol/L	1,614 (548)	4.79 (4.54**–**5.04)	1,953 (693)	4.81 (4.56**–**5.05)	0.175
	sUA, μmol/L	1,672 (490)	282 (245**–**329)	1,989 (657)	290 (250**–**340)	**0.003**
Males	Age, years	1,307 (0)	9.0 (6.1**–**12)	1,588 (0)	8.6 (5.8**–**11.8)	**0.01**
	zBMI	1,307 (0)	−0.25 (−1.06**–**0.76)	1,588 (0)	0.01 (−0.79**–**1.12)	**<0.001**
	WHtR	1,285 (22)	0.3 (0.4**–**0.47)	1,056 (532)	0.44 (0.4**–**0.48)	0.07
	SBP, mmHg	1,254 (53)	102 (94**–**111)	1,545 (43)	102 (93**–**110)	0.465
	DBP, mmHg	1,254 (53)	62 (56**–**68)	1,545 (43)	62 (55**–**68)	0.302
	Hb, g/L	1,299 (8)	135 (129**–**142)	1,518 (70)	134 (127**–**141)	**0.013**
	TG, mmol/L	1,047 (260)	0.76 (0.6**–**1.02)	1,252 (336)	0.68 (0.53**–**0.92)	**<0.001**
	TC, mmol/L	1,047 (260)	4.12 (3.69**–**4.64)	1,252 (336)	4.32 (3.87**–**4.87)	**<0.001**
	LDL-C, mmol/L	1,003 (304)	2.12 (1.82**–**2.46)	1,234 (354)	2.44 (2.09**–**2.83)	**<0.001**
	HDL-C, mmol/L	1,003 (304)	1.45 (1.27**–**1.64)	1,234 (354)	1.47 (1.29**–**1.68)	**0.038**
	25(OH)D, nmol/L	1,047 (260)	52.8 (46.4**–**60.3)	1,168 (420)	47.3 (39.2**–**57.7)	**<0.001**
	Fasting glucose, mmol/L	1,003 (301)	4.85 (4.59**–**5.08)	1,221 (367)	4.84 (4.61**–**5.1)	0.68
	sUA, μmol/L	1,047 (260)	286 (248**–**335)	1,240 (348)	293 (253**–**349)	**0.006**
Females	Age, years	855 (0)	8.6 (6**–**12)	1,058 (0)	8.1 (5.6**–**11.9)	**0.009**
	zBMI	855 (0)	−0.41 (−1.08**–**0.36)	1,058 (0)	−0.31 (−1.03**–**0.48)	0.052
	WHtR	836 (19)	0.42 (0.39**–**0.45)	728 (330)	0.42 (0.39**–**0.46)	0.712
	SBP, mmHg	826 (29)	100 (93**–**109)	1,034 (24)	100 (93**–**108)	0.775
	DBP, mmHg	826 (29)	62 (56**–**69)	1,034 (24)	62 (56**–**68)	0.287
	Hb, g/L	847 (8)	134 (128**–**139)	990 (68)	132 (126**–**137)	**<0.001**
	TG, mmol/L	625 (230)	0.8 (0.65**–**1.06)	768 (290)	0.73 (0.58**–**0.98)	**<0.001**
	TC, mmol/L	625 (230)	4.21 (3.77**–**4.73)	768 (290)	4.39 (3.95**–**4.87)	**<0.001**
	LDL-C, mmol/L	600 (255)	2.21 (1.9**–**2.6)	749 (309)	2.53 (2.16**–**2.93)	**<0.001**
	HDL-C, mmol/L	600 (255)	1.4 (1.25**–**1.58)	749 (309)	1.42 (1.26**–**1.61)	0.075
	25(OH)D, nmol/L	627 (228)	51 (43.3**–**59)	712 (346)	46 (37**–**56)	**<0.001**
	Fasting glucose, mmol/L	608 (247)	4.71 (4.48**–**4.94)	732 (326)	4.74 (4.5**–**4.98)	0.104
	sUA, μmol/L	625 (230)	274 (242**–**319)	749 (309)	285 (247**–**331)	**0.012**

M, median; P25, the 25^th^ percentile; P75, the 75^th^ percentile; zBMI, z score for body mass index; WHtR, weight to height ratio; SBP, systolic blood pressure; DBP, diastolic blood pressure; Hb, hemoglobin; TG, triglycerides; TC, total cholesterol; LDL-C, low density lipoprotein cholesterol; HDL-C, high density lipoprotein cholesterol; sUA, serum uric acid.

^*^n refers to the number of children who did not have certain measurement or test results. The values in bold indicating that *P* < 0.05.

Table 3 presents the results of quantile regression models, it was found that BMI, TC, LDL-C, and sUA were positively correlated with year, while Hb, TG, and 25(OH)D were negatively associated with year (all *P* < 0.05) after adjusting for age, sex, and BMI when appropriate. The differences in WHtR, SBP, DBP, HDL-C and fasting glucose between 2019 and 2020 were not statistically significant (all *P* > 0.05).

**Table 3:** Quantile regression analyses to assess the impact of lockdown after the COVID-19 outbreak on children's physiological health indicators.

**Health indicators**	*N* (n^*^)	**Year (2020 vs. 2019)**		
		β	**SE**	***P***
BMI, kg/m^2^	4,808 (0)	0.302	0.091	**0.001**
WHtR	3,905 (903)	0.001	0.002	0.515
SBP, mmHg	4,659 (149)	0.572	0.341	0.093
DBP, mmHg	4,659 (149)	0.135	0.282	0.632
Hb, g/L	4,654 (154)	−1.006	0.298	**0.001**
TG, mmol/L	3,692 (1,116)	−0.071	0.012	**<0.001**
TC, mmol/L	3,692 (1,116)	0.177	0.029	**<0.001**
LDL-C, mmol/L	3,586 (1,222)	0.294	0.023	**<0.001**
HDL-C, mmol/L	3,586 (1,222)	0.018	0.012	0.147
25(OH)D, nmol/L	3,554 (1,254)	−6.05	0.453	**<0.001**
Blood glucose, mmol/L	3,567 (1,241)	0.029	0.015	**0.044**
sUA, μmol/L	3,661 (1,147)	9.754	2.739	**<0.001**

BMI, body mass index; WHtR, weight to height ratio; SBP, systolic blood pressure; DBP, diastolic blood pressure; Hb, Hemoglobin; TG, triglycerides; TC, total cholesterol; LDL-C, low density lipoprotein cholesterol; HDL-C, high density lipoprotein cholesterol; sUA, serum uric acid.

Age, sex and BMI were adjusted in all models, when BMI and WHtR ratio are the dependent variables, only age and sex were adjusted in the models.

^*^n refers to the number of children who did not have certain measurement or test results. The values in bold indicating that *P* < 0.05.

Table 4 shows the prevalence of abnormal health conditions in both male and female children examined in 2019 and 2020, respectively. The prevalence of OW and OB, hTC, hLDL-C, vitamin D deficiency, and hUA were 20.6, 16.2, 10, 22.6, and 18.9%, respectively; and were higher in children examined in 2020 than those in 2019 (all *P* < 0.05). Meanwhile, the prevalence of hTG was slightly lower in 2020 than in 2019 (*P* < 0.05). As for the prevalence of abdominal obesity, hypertension, hyperglycemia, anemia and low HDL-C, no significant differences were identified in children examined in 2020 and 2019 (*P* > 0.05). Additionally, we found that the prevalence of vitamin D deficiency increased in both males (from 5.6 to 20.5%) and females (from 12.1 to 26.1%). Moreover, anemia and hTG prevalence decreased mainly in boys, while hUA prevalence increased mainly in girls.

**Table 4:** Abnormal health conditions before and after COVID-19 outbreak among children aged 3~18 years' old.

**Characteristics**	**Total**			**Male**			**Female**		
	**2019**	**2020**	***P***	**2019**	**2020**	***P***	**2019**	**2020**	***P***
	***N*** **(%)**	***N*** **(%)**		***N*** **(%)**	***N*** **(%)**		***N*** **(%)**	***N*** **(%)**	
**zBMI**			**0.002**			**0.003**			**0.046**
Underweight	144 (6.7)	135 (5.1)		92 (7.1)	83 (5.2)		52 (6.1)	52 (4.9)	
Normal weight	1,657 (76.6)	1,966 (74.3)		937 (71.7)	1,099 (69.2)		720 (84.2)	867 (82)	
Overweight	245 (11.3)	332 (12.5)		177 (13.5)	229 (14.4)		68 (7.9)	103 (9.7)	
Obesity	16 (5.4)	213 (8.1)		101 (7.7)	177 (11.2)		15 (1.8)	36 (3.4)	
**Central obesity, yes**	361 (16.7)	545 (20.6)	0.274	273 (21.3)	248 (23.5)	0.195	107 (12.8)	96 (13.2)	0.82
**Hypertension, yes**	165 (7.9)	221 (8.6)	0.433	92 (7.3)	133 (8.6)	0.218	73 (8.8)	88 (8.5)	0.803
**Anemia, yes**	38 (1.8)	38 (1.5)	0.493	23 (1.8)	12 (0.8)	**0.019**	15 (1.8)	26 (2.6)	0.216
**hTG, yes**	71 (4.3)	57 (2.8)	**0.018**	49 (4.7)	33 (2.6)	**0.008**	22 (3.5)	24 (3.1)	0.682
**hTC, yes**	170 (10.2)	327 (16.2)	**<0.001**	94 (9)	206 (16.5)	**<0.001**	76 (12.2)	121 (15.8)	0.055
**hLDL-C, yes**	47 (2.9)	198 (10)	**<0.001**	22 (2.2)	116 (9.4)	**<0.001**	25 (4.2)	82 (10.9)	**<0.001**
**lHDL-C, yes**	79 (4.9)	73 (3.7)	0.065	49 (4.9)	43 (3.5)	0.097	30 (5)	30 (4)	0.378
**Vitamin D**			**<0.001**			**<0.001**			**<0.001**
Deficiency	135 (8.1)	425 (22.6)		59 (5.6)	239 (20.5)		76 (12.1)	186 (26.1)	
Insufficient	556 (33.2)	700 (37.2)		338 (32.3)	446 (38.2)		218 (34.8)	254 (35.7)	
Sufficient	983 (58.7)	755 (40.2)		650 (62.1)	483 (41.3)		333 (53.1)	272 (38.2)	
**Hyperglycemia, yes**	18 (1.1)	29 (1.5)	0.335	13 (1.3)	23 (1.9)	0.271	5 (0.8)	6 (0.8)	0.996
**hUA, yes**	252 (15.1)	376 (18.9)	**0.002**	194 (18.6)	270 (21.8)	0.055	58 (9.3)	106 (14.2)	**0.005**

zBMI, z score for body mass index; hTG, hypertriglycerides; hTC, high total cholesterol; hLDL-C, high low density lipoprotein cholesterol; lHDL-C, low high density lipoprotein cholesterol; hUA, high uric acid.

^*^The numbers of children who had no certain measurement or test results were the same as in Table 2 and were not presented here. The values in bold indicating that *P* < 0.05.

## Discussion

In this retrospective study using real-world evidence from the Children's Hospital, we found that after at least 3 months of lockdown, children were more likely to be OW/OB, have higher serum TC, LDL-C, fasting glucose and UA levels, and more likely to be vitamin D deficient and anemia compared with their counterparts from the same period in 2019. Meanwhile, the TG level and prevalence of high TG were slightly lower in 2020.

The increasing prevalence of OW and OB in children after the COVID-19 outbreak we found in our study was consistent with previous studies conducted in other countries (18–22). In European countries such as Spain and Poland (18, 19), 25.8~35% of children's body weight increased during COVID-19 lockdown. In Palestine (20), as high as 41.7% of adolescents gained more weight. In the United States, zBMI of children has increased by 0.056 after 2 months of school closure (21). The situation of children's health status during the lockdown in China was not exceptional (22). In a cohort study conducted in Henan, China (22), 28.1% of children aged 7~12 years with normal weight before the COVID-19 outbreak developed into OW or OB, while 42.4% of OW children before COVID-19 developed into OB.

Poor dietary choices, more sedentary and screen time as well as less physical activity might be the explanation for children's weight gaining during the COVID-19 lockdown. In a retrospective survey including 10,082 school-aged youths from 31 provinces in China (7), increases in consumption of wheat products, staple foods, and preserved vegetables were observed. Poor diet quality during COVID-19 lockdown was reported to be associated with an increase in BMI (19). Moreover, after the implementation of the “classes suspended, learning continues” policy during the lockdown in China, e-learning became the only-option for school-aged children and adolescents, which inevitably led to higher exposure to digital screens and increased time of sedentary behaviors. In a study performed in Shanghai, China, screen time in 7–12 years old children has increased from 0.67 h/day before the COVID-19 outbreak to 5.24 h afterwards (8). Androutsos et al. found that the prevalence of children having excessive screen time, i.e., ≥2 h/day, increased from 23.4% before lockdown to 68.4% after, and 66.9% children reported decreased time spent in physical activity (9).

We have also observed an increase in TC, LDL-C, fasting glucose and sUA levels and a decrease in TG level after the COVID-19 outbreak in our study, similar with two other findings (23, 24). In one study carried out in around 100 Korean children with paired data on lipid parameters before and after the COVID lockdown (23), the average levels of LDL-C and uric acid increased by 6.2 and 0.3 mg/dL during the COVID-19 period, respectively. In another study performed in 312 children with type 1 diabetes in India (24), children with dyslipidemia increased from 30% before COVID outbreak to 51% during COVID restrictions.

Apart from the decreasing TG, all the other changes, including higher BMI, indicated metabolic disorder. It was reported that (25) even a reduction in daily physical activity of 3 days influenced glycemic control, and physical inactivity of 2 weeks influenced lipid profile and lean mass, especially in overweight participants. Inconsistent with the two afore-mentioned studies (23, 24). TG levels in children examined in 2020 decreased in our study. Though we did not collect dietary intake and physical activity information in the present study, it is well-established that increasing consumption of saturated fatty acid and low-quality carbohydrates can result in increasing cholesterols, while decreasing energy contribution from carbohydrates can lower TG level (26, 27). In a recent study, unfavorable dietary changes such as increases in fat/oil intake and decreases in fruit intake during the 2020 COVID-19 pandemic were reported (28). However, researches on changes of energy source and distribution during lockdown are still limited and more profound studies are warranted.

A higher prevalence of vitamin D deficiency after months of lockdown in 2020 was also identified in our study, consistent with findings in the Korean children (23). The decrease of 25(OH)D levels in children might be caused by inadequate sunlight exposure due to less outdoor physical activity during the lockdown period. Additionally, a greater increase of vitamin D deficiency was found in boys than in girls, which may be explained by higher exposure to sunlight in boys than in girls before the COVID-19 pandemic (15, 29) and an undifferentiated reduction of sun exposure due to the lockdown. As a crucial nutrient in children's bone health and a protective factor for metabolic health, adequate vitamin D consumption should be considered, especially when sufficient sun exposure was not available.

Our study did not confirm significant changes in children's blood pressure. Inconsistently, in a study performed in 445 children from Henan, China (22), 46.6 % of children with normal blood pressure before the COVID-19 outbreak turned to be children with pre- or elevated blood pressure, and the prevalence of hypertension increased from 20.9% at baseline to 34.6%. However, the sample size in this study was relatively smaller, and only children aged 7~12 years were included. Besides, the percentage of children with OW/OB (65.6%) before the outbreak in this study was much higher than that in our study (16.7%). It was proved that OB was significantly associated with an increased risk of essential hypertension (30).

Although there are lots of studies on the adverse impact of COVID-19 on children's lifestyle change, our study was one of the few pieces of real-world evidence that using hospital-based health records to investigate the impacts of COVID-19 lockdown on children's physiological health. By doing so, we avoided recall bias because all anthropometric parameters were measured by well-trained health professionals and the laboratory indexes were detected according to established standards. In addition, our study has included an extensive set of health indicators such as weight, height, waist circumference, systolic and diastolic blood pressure, lipid parameters, serum 25(OH)D level, sUA, and fasting blood glucose, which can comprehensively reflect the metabolism of children.

The study has several limitations warranting for attentions as well. First, selection bias may exist since all subjects were from hospitals but not communities, which may limit the generalization of the study conclusion. Second, children having their health checkups in 2020 were slightly younger than those in 2019, which might compromise the homogeneity of comparison. However, we have adjusted age inregression models. Thus, findings in our study may not be mistakenly estimated. Third, we did not collect information on children's diets, 24-h physical activity or adverse events occurred during COVID-19 pandemic. Although further analysis on factors associated with physiological change was infeasible, insufficient understanding of these contributing factors should have little change on our conclusion.

## Conclusions

In this real-world study, we observed adverse changes in children's weight status, cholesterols, sUA and vitamin D levels after months of COVID-19 lockdown. Since childhood obesity and metabolic abnormality were proved to be risk factors for future cardiovascular diseases and type 2 diabetes (31), we call for attention from health professionals, educational practitioners, policymakers, and other stake-holders as well as parents on this issue. As reported by UNICEF, 91% of students worldwide – around 1.6 billion children and young people were affected by the COVID-19 pandemic (32). Considering physical inactivity pandemic is getting worse (33), we recommend a more nutrition-balanced diet and sufficient physical activity for children to keep healthy and fit in this new normal against COVID-19.

## Data availability statement

The raw data supporting the conclusions of this article will be made available by the authors upon request, without undue reservation.

## Ethics statement

The studies involving human participants were reviewed and approved by the Medical Ethics Committee of Children's Hospital, Zhejiang University School of Medicine. Written informed consent for participation was not provided by the participants' legal guardians/next of kin because: Data used in this study was retrieved from electronic records in the hospital. By the time this study was conducted, all participants have finished their health checkup and left the hospital. Considering no additional physical harm were added to the participants due to this study, informed consent was waived by the Medical Ethics Committee of Children's Hospital, Zhejiang University School of Medicine [Review number: 2021-IRB-185].

## Author contributions

WHD, TMG, GNB, and JS: study concept, design, and drafting of the manuscript. WHD, TMG, BQZ, and YS: acquisition of data. WHD, TMG, BQZ, YS, XYH, GNB, and JS: statistical analysis, interpretation of data, and critical revision of the manuscript for important intellectual content. JS: obtaining funding and study supervision. BQZ and JS: administrative, technical, or material support. All authors contributed to the article and approved the submitted version.
